# Gene model for the ortholog of *Ptp61F* in *Drosophila ananassae*

**DOI:** 10.17912/micropub.biology.001002

**Published:** 2026-07-06

**Authors:** Anne E. Backlund, Logan Cohen, Priya Vaid, Chloe Nason, Kara Kneuper, Ali Jade Miller, Christopher E. Ellison, Lindsey J. Long, Daron Barnard, Chinmay P. Rele, Laura K. Reed

**Affiliations:** 1 The University of Alabama, Tuscaloosa, AL USA; 2 Worcester State University, Worcester MA, USA; 3 Rutgers University, New Brunswick, NJ USA; 4 Oklahoma Christian University, Edmond, OK USA

## Abstract

Gene model for the ortholog of *Protein tyrosine phosphatase 61F* ( *Ptp61F* ) in the *Drosophila ananassae* May 2011 (Agencourt dana_caf1/DanaCAF1) Genome Assembly (GenBank Accession: GCA_000005115.1). This ortholog was characterized as part of a developing dataset to study the evolution of the Insulin/insulin-like growth factor signaling pathway (IIS) across the genus *Drosophila* using the Genomics Education Partnership gene annotation protocol for Course-based Undergraduate Research Experiences.

**Figure 1. Genomic neighborhoods for Ptp61F in Drosophila melanogaster and Drosophila ananassae:** **
(A) Synteny comparison of the genomic neighborhoods for *Ptp61F* in *Drosophila melanogaster* and *Drosophila ananassae* .** Thin underlying arrows indicate the DNA strand within which the gene– *Ptp61F* –is located in *D. melanogaster* (top) and *D. ananassae* (bottom). The thin arrow pointing to the right indicates that *Ptp61F* is on the positive (+) strand in *Drosophila ananassae* , and the thin arrow pointing to the left indicates that *Ptp61F* is on the negative (-) strand in *D. melanogaster* . The wide gene arrows pointing in the same direction as *Ptp61F* are on the same strand relative to the thin underlying arrows, while wide gene arrows pointing in the opposite direction of *Ptp61F* are on the opposite strand relative to the thin underlying arrows. White gene arrows in *Drosophila ananassae* indicate orthology to the corresponding gene in *D. melanogaster* , while black gene arrows indicate non-orthology. Gene symbols given in the *Drosophila ananassae* gene arrows indicate the orthologous gene in *D. melanogaster* , while the locus identifiers are specific to *Drosophila ananassae* . **(B) Gene Model in GEP UCSC Track Data Hub** (Raney et al., 2014). The coding-regions of *Ptp61F* in *Drosophila ananassae* are displayed in the User Supplied Track (black); coding CDSs are depicted by thick rectangles and introns by thin lines with arrows indicating the direction of transcription. Subsequent evidence tracks include BLAT Alignments of NCBI RefSeq Genes (dark blue, alignment of Ref-Seq genes for *Drosophila ananassae* ), Spaln of *D. melanogaster* Proteins (purple, alignment of Ref-Seq proteins from *D. melanogaster* ), Transcripts and Coding Regions Predicted by TransDecoder (dark green), RNA-Seq from Adult Females, Adult Males, and *Wolbachia* -cured Embryos (red, light blue, and pink, respectively; alignment of Illumina RNA-Seq reads from *Drosophila ananassae* ), and Splice Junctions Predicted by regtools using *Drosophila ananassae* RNA-Seq (SRP006203, SRP007906, PRJNA257286, PRJNA388952). Splice junctions shown have a minimum read-depth of 10 with 50-99, 100-499, 500-999, >1000 supporting reads in green, pink, brown, and red, respectively. **(C) Dot Plot of Ptp61F-PA in *D. melanogaster* ( *x* -axis) vs. the orthologous peptide in *Drosophila ananassae* ( *y* -axis).** Amino acid number is indicated along the left and bottom; coding-CDS number is indicated along the top and right, and CDSs are also highlighted with alternating colors. Line breaks in the dot plot indicate mismatching amino acids at the specified location between species. **(D)** **Gene Model in GEP UCSC Track Data Hub** (Raney et al., 2014). The same evidence tracks as Figure 1B are shown in this image. We hypothesize that the isoform Ptp61F-PC does not exist in *D. ananassae* . In addition, we hypothesize that there is a novel isoform, Ptp61F-PNF.

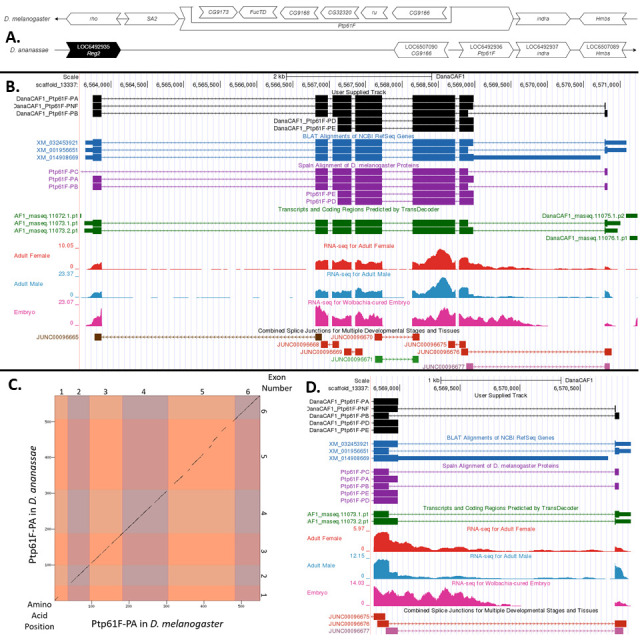



## Description

**Table:** 

* This article reports a predicted gene model generated by undergraduate work using a structured gene model annotation protocol defined by the Genomics Education Partnership (GEP; thegep.org ) for Course-based Undergraduate Research Experience (CURE). The following information in this box may be repeated in other articles submitted by participants using the same GEP CURE protocol for annotating Drosophila species orthologs of Drosophila melanogaster genes in the insulin signaling pathway. * "In this GEP CURE protocol students use web-based tools to manually annotate genes in non-model *Drosophila* species based on orthology to genes in the well-annotated model organism fruitfly *Drosophila melanogaster* . The GEP uses web-based tools to allow undergraduates to participate in course-based research by generating manual annotations of genes in non-model species (Rele et al., 2023). Computational-based gene predictions in any organism are often improved by careful manual annotation and curation, allowing for more accurate analyses of gene and genome evolution (Mudge and Harrow 2016; Tello-Ruiz et al., 2019). These models of orthologous genes across species, such as the one presented here, then provide a reliable basis for further evolutionary genomic analyses when made available to the scientific community.” (Myers et al., 2024). “The particular gene ortholog described here was characterized as part of a developing dataset to study the evolution of the Insulin/insulin-like growth factor signaling pathway (IIS) across the genus *Drosophila* . The Insulin/insulin-like growth factor signaling pathway (IIS) is a highly conserved signaling pathway in animals and is central to mediating organismal responses to nutrients (Hietakangas and Cohen 2009; Grewal 2009).” (Myers et al., 2024). “ *D* . *ananassae* (NCBI:txid7217) is part of the *melanogaster* species group within the subgenus *Sophophora* of the genus *Drosophila* (Sturtevant 1939; Bock and Wheeler 1972). It was first described by Doleschall (1858). *D. ananassae* is circumtropical (Markow and O'Grady 2005; https://www.taxodros.uzh.ch, accessed 1 Feb 2023), and often associated with human settlement (Singh 2010). It has been extensively studied as a model for its cytogenetic and genetic characteristics, and in experimental evolution (Kikkawa 1938; Singh and Yadav 2015).” (Lawson et al., 2024).

We propose a gene model for the *Drosophila ananassae* ortholog of the *D. melanogaster* *Protein tyrosine phosphatase 61F* ( *Ptp61F* ) gene. The genomic region of the ortholog corresponds to the uncharacterized protein XP_014764155.1 (Locus ID LOC6492936 ) in the May 2011 (Agencourt dana_caf1/DanaCAF1) Genome Assembly of *Drosophila ananassae* ( GCA_000005115.1 , Drosophila 12 Genomes Consortium et al., 2007). This model is based on RNA-Seq data from *Drosophila ananassae* ( SRP006203 , SRP007906 , PRJNA257286 , PRJNA388952 ; Graveley et al., 2011) and *Ptp61F* in *D. melanogaster* using FlyBase release FB2023_03 ( GCA_000001215.4 ) (Larkin et al., 2021; Gramates et al., 2022; Jenkins et al., 2022).

The protein product of *Ptp61F* (PTP61) negatively regulates the Insulin/TOR pathway by dephosphorylating the insulin receptor (InR) (Wu et al., 2011; Buszard et al., 2013). PTP61Fm-mediated dephosphorylation of InR requires an interaction with the SH2/SH3 adaptor protein Dock ( *dreadlocks* ) while PTP61Fn dephosphorylates InR in a Dock-independent manner (Clemens et al., 1996; Buszard et al., 2013; Willoughby et al., 2017). The *Ptp61F* gene in *Drosophila melanogaster* was first isolated using rat PTP61F cDNA in a low stringency hybridization screening method (McLaughlin and Dixon 1993). *Ptp61F* is differentially spliced where the longer isoform (PTP61Fm) is targeted to the ER by the hydrophobic C-terminal domain, and the shorter isoform (PTP61Fn) is targeted to the nucleus by a nuclear localization signal (NLS) (McLaughlin and Dixon 1993). PTP61F plays a role in the regulation of a variety of signaling pathways through negative regulation of tyrosine kinases including Janus kinase/signal transducers and activators of transcription (JAK/STAT), Mitogen-activated protein kinase (MAPK), epidermal growth factor receptor (EGFR), and platelet-derived growth factor/vascular endothelial growth factor receptor (PVR) (Baeg et al., 2005; Buszard et al., 2013; Tchankouo-Nguetcheu et al., 2014; Willoughby et al., 2017).

***Synteny***

The reference gene, *Ptp61F ,* occurs on chromosome 3L in *D. melanogaster* and is flanked upstream by *rhomboid* ​( *rho* ) and *Stromalin 2* ( *SA2* ) and downstream by *indra* and *Hydroxymethylbilane synthase* ( *Hmbs* ). There are also six genes nested within *Ptp61F* in *D. melanogaster* : *CG9173 , FucTD , CG9168 , CG32320 , ru ,* and *CG9166 .* The *tblastn* search of *D. melanogaster* Ptp61F-PA (query) against the *Drosophila ananassae* (GenBank Accession: GCA_000005115.1 ) Genome Assembly (database) placed the putative ortholog of *Ptp61F* within scaffold_13337 at locus LOC6492936 ( XP_014764155.1 )— with an E-value of 7e-28 and a percent identity of 84.48%. Furthermore, the putative ortholog is flanked upstream by LOC6492935 ( XP_001956685.1 ) and LOC6507090 ( XP_001956686.1 ), which correspond to *Reg-2* and *CG9166* in *D. melanogaster* (E-value: 0.0 and 2e-140; identity: 89.62% and 83.55%, respectively, as determined by *blastp* ) ( Figure 1A ; Altschul et al., 1990). The putative ortholog of *Ptp61F* is flanked downstream by LOC6492937 (XP_ 001956688.1) and LOC6507089 (XP_ 001956689.1), which correspond to *indra* and *Hmbs* in *D. melanogaster* (E-value: 2e-96 and 0.0; identity: 32.20% and 86.85%, respectively, as determined by *blastp* ). The putative ortholog assignment for *Ptp61F* in *D. ananassae* is supported by the following evidence: The *blastp* and *tblastn* results support the presence of a *Ptp61F* ortholog in this location in *D. ananassae* although synteny is not completely conserved. In *D. melanogaster* , the Ptp61F-PC isoform has 6 genes nested within it, but this characteristic is not present in *D. ananassae* , so we hypothesize that the Ptp61F-PC isoform does not exist in this species. *CG9166* is present in both genomic neighborhoods, but in slightly different locations (i.e., not nested within *Ptp61F* ), and the first and second downstream genes ( *indra* and *Hmbs* ) are orthologous. Therefore, we conclude that LOC6492936 is the correct ortholog of *Ptp61F* in *D. ananassae* ( Figure 1A ).

***Protein Model***

*Ptp61F* in *D. melanogaster* has mRNA isoforms: Ptp61F-RA, Ptp61F-RB, Ptp61F-RC, Ptp61F-RD, and Ptp61F-RE. Ptp61F-RE and Ptp61F-RD have identical coding sequences. mRNA soform Ptp61F-RA contains six CDSs, Ptp61F-RB and Ptp61F-RC have seven CDSs, and Ptp61F-RD and Ptp61F-RE have four CDSs. In *D. ananassae* , the isoform count is not conserved (see Special characteristics of the protein model), and we predict five total isoforms (Ptp61F-PA, Ptp61F-PB, Ptp61F-PD, Ptp61F-PE,and Ptp61F-PNF) *.* The sequence of Ptp61F-PA in *Drosophila ananassae* has 76.87% identity (E-value: 0.0) with the protein-coding isoform Ptp61F-PA in *D. melanogaster* , as determined by *blastp* ( Figure 1C ). Given that *D. ananassae* and *D. melanogaster* belong to distinct species groups, the observed degree protein divergence is well explained and consistent with that seen in other ortholog comparisons between these two species ( *Drosophila* 12 Genomes Consortium et al., 2007). Coordinates of this curated gene model are stored by NCBI at GenBank/BankIt (accessions BK064622 , BK064623 , BK064624 , BK064625 , and BK064626 ). These data are also archived in the CaltechDATA repository (see “Extended Data” section below).

***Special characteristics of the protein model***

We hypothesize that the isoform Ptp61F-PC does not exist in *D. ananassae* . In addition, we hypothesize that there is a new isoform, Ptp61F-PNF, due to alternative splicing. Ptp61F-PC in *D. melanogaster* has a long first intron that has six genes nested within it, but this does not appear in *D. ananassae* ( Figure 1A ). A *tblastn* search of the amino acid sequence of the first CDS in Ptp61F-PC in *D. melanogaster* against the *D. ananassae* genome did not return any results, and there are no predicted splice junctions consistent with a long first intron for Ptp61F-PC in this species. This leads us to conclude that the Ptp61F-PC isoform is not present in *D. ananassae* . Finally, we determined that XM_032453921 ( LOC6492936 ) is a novel isoform, as there is not an orthologous isoform in *D. melanogaster* that has the same gene structure. It has seven CDSs, and its sixth CDS is longer than the corresponding CDS in isoform Ptp61F-PB ( Figure 1D ). The seventh CDS of the proposed novel isoform is only eight base pairs long (including the stop codon), as compared to 37 base pairs long for the seventh CDS of isoform Ptp61F-RB. The sixth and seventh CDSs of this novel isoform are supported by a splice junction with a score of 457 ( Figure 1D, shown in pink). These features of XM_032453921 ( LOC6492936 ) lead us to believe that new alternative splicing has occurred, resulting in a novel isoform. This novel isoform has 75.86% protein identity (E-value: 0.0) to the *D. melanogaster* Ptp61F-PB isoform. We propose that this isoform be named Ptp61F-PNF.

## Methods

Detailed methods including algorithms, database versions, and citations for the complete annotation process can be found in Rele et al. (2023). Briefly, students use the GEP instance of the UCSC Genome Browser v.435 ( https://gander.wustl.edu ; Kent WJ et al., 2002; Navarro Gonzalez et al., 2021) to examine the genomic neighborhood of their reference IIS gene in the *D. melanogaster* genome assembly (Aug. 2014; BDGP Release 6 + ISO1 MT/dm6). Students then retrieve the protein sequence for the *D. melanogaster* reference gene for a given isoform and run it using *tblastn* against their target *Drosophila* species genome assembly on the NCBI BLAST server ( https://blast.ncbi.nlm.nih.gov/Blast.cgi ; Altschul et al., 1990) to identify potential orthologs. To validate the potential ortholog, students compare the local genomic neighborhood of their potential ortholog with the genomic neighborhood of their reference gene in *D. melanogaster* . This local synteny analysis includes at minimum the two upstream and downstream genes relative to their putative ortholog. They also explore other sets of genomic evidence using multiple alignment tracks in the Genome Browser, including BLAT alignments of RefSeq Genes, Spaln alignment of *D. melanogaster* proteins, multiple gene prediction tracks (e.g., GeMoMa, Geneid, Augustus), and modENCODE RNA-Seq from the target species. Detailed explanation of how these lines of genomic evidenced are leveraged by students in gene model development are described in Rele et al. (2023). Genomic structure information (e.g., CDSs, intron-exon number and boundaries, number of isoforms) for the *D. melanogaster* reference gene is retrieved through the Gene Record Finder ( https://gander.wustl.edu/~wilson/dmelgenerecord/index.html ; Rele et al *.,* 2023). Approximate splice sites within the target gene are determined using *tblastn* using the CDSs from the *D. melanogaste* r reference gene. Coordinates of CDSs are then refined by examining aligned modENCODE RNA-Seq data, and by applying paradigms of molecular biology such as identifying canonical splice site sequences and ensuring the maintenance of an open reading frame across hypothesized splice sites. Students then confirm the biological validity of their target gene model using the Gene Model Checker ( https://gander.wustl.edu/~wilson/genechecker/index.html ; Rele et al., 2023), which compares the structure and translated sequence from their hypothesized target gene model against the *D. melanogaster* reference gene model. At least two independent models for a gene are generated by students under mentorship of their faculty course instructors. Those models are then reconciled by a third independent researcher mentored by the project leaders to produce the final model. Note: comparison of 5' and 3' UTR sequence information is not included in this GEP CURE protocol (Gruys et al., 2025).

## Data Availability

Description: Zip file containing FASTA, PEP, and GFF. Resource Type: Model. DOI: https://doi.org/10.22002/7tpn6-87t55

## References

[R1] Altschul SF, Gish W, Miller W, Myers EW, Lipman DJ (1990). Basic local alignment search tool.. J Mol Biol.

[R2] Baeg GH, Zhou R, Perrimon N (2005). Genome-wide RNAi analysis of JAK/STAT signaling components in Drosophila.. Genes Dev.

[R3] Bock IR, Wheeler MR. (1972). The Drosophila melanogaster species group. Univ. Texas Publs Stud. Genet. 7(7213): 1-102. FBrf0024428

[R4] Buszard BJ, Johnson TK, Meng TC, Burke R, Warr CG, Tiganis T (2013). The nucleus- and endoplasmic reticulum-targeted forms of protein tyrosine phosphatase 61F regulate Drosophila growth, life span, and fecundity.. Mol Cell Biol.

[R5] Clemens JC, Ursuliak Z, Clemens KK, Price JV, Dixon JE (1996). A Drosophila protein-tyrosine phosphatase associates with an adapter protein required for axonal guidance.. J Biol Chem.

[R6] Doleschall CL. 1858. Derde bijdrage tot de kennis der Dipteren fauna van nederlandsch indie. Natuurk. Tijd. Ned.-Indie 17: 73-128. FBrf0000091

[R7] Clark AG, Eisen MB, Smith DR, Bergman CM, Oliver B, Markow TA, Kaufman TC, Kellis M, Gelbart W, Iyer VN, Pollard DA, Sackton TB, Larracuente AM, Singh ND, Abad JP, Abt DN, Adryan B, Aguade M, Akashi H, Anderson WW, Aquadro CF, Ardell DH, Arguello R, Artieri CG, Barbash DA, Barker D, Barsanti P, Batterham P, Batzoglou S, Begun D, Bhutkar A, Blanco E, Bosak SA, Bradley RK, Brand AD, Brent MR, Brooks AN, Brown RH, Butlin RK, Caggese C, Calvi BR, Bernardo de Carvalho A, Caspi A, Castrezana S, Celniker SE, Chang JL, Chapple C, Chatterji S, Chinwalla A, Civetta A, Clifton SW, Comeron JM, Costello JC, Coyne JA, Daub J, David RG, Delcher AL, Delehaunty K, Do CB, Ebling H, Edwards K, Eickbush T, Evans JD, Filipski A, Findeiss S, Freyhult E, Fulton L, Fulton R, Garcia AC, Gardiner A, Garfield DA, Garvin BE, Gibson G, Gilbert D, Gnerre S, Godfrey J, Good R, Gotea V, Gravely B, Greenberg AJ, Griffiths-Jones S, Gross S, Guigo R, Gustafson EA, Haerty W, Hahn MW, Halligan DL, Halpern AL, Halter GM, Han MV, Heger A, Hillier L, Hinrichs AS, Holmes I, Hoskins RA, Hubisz MJ, Hultmark D, Huntley MA, Jaffe DB, Jagadeeshan S, Jeck WR, Johnson J, Jones CD, Jordan WC, Karpen GH, Kataoka E, Keightley PD, Kheradpour P, Kirkness EF, Koerich LB, Kristiansen K, Kudrna D, Kulathinal RJ, Kumar S, Kwok R, Lander E, Langley CH, Lapoint R, Lazzaro BP, Lee SJ, Levesque L, Li R, Lin CF, Lin MF, Lindblad-Toh K, Llopart A, Long M, Low L, Lozovsky E, Lu J, Luo M, Machado CA, Makalowski W, Marzo M, Matsuda M, Matzkin L, McAllister B, McBride CS, McKernan B, McKernan K, Mendez-Lago M, Minx P, Mollenhauer MU, Montooth K, Mount SM, Mu X, Myers E, Negre B, Newfeld S, Nielsen R, Noor MA, O'Grady P, Pachter L, Papaceit M, Parisi MJ, Parisi M, Parts L, Pedersen JS, Pesole G, Phillippy AM, Ponting CP, Pop M, Porcelli D, Powell JR, Prohaska S, Pruitt K, Puig M, Quesneville H, Ram KR, Rand D, Rasmussen MD, Reed LK, Reenan R, Reily A, Remington KA, Rieger TT, Ritchie MG, Robin C, Rogers YH, Rohde C, Rozas J, Rubenfield MJ, Ruiz A, Russo S, Salzberg SL, Sanchez-Gracia A, Saranga DJ, Sato H, Schaeffer SW, Schatz MC, Schlenke T, Schwartz R, Segarra C, Singh RS, Sirot L, Sirota M, Sisneros NB, Smith CD, Smith TF, Spieth J, Stage DE, Stark A, Stephan W, Strausberg RL, Strempel S, Sturgill D, Sutton G, Sutton GG, Tao W, Teichmann S, Tobari YN, Tomimura Y, Tsolas JM, Valente VL, Venter E, Venter JC, Vicario S, Vieira FG, Vilella AJ, Villasante A, Walenz B, Wang J, Wasserman M, Watts T, Wilson D, Wilson RK, Wing RA, Wolfner MF, Wong A, Wong GK, Wu CI, Wu G, Yamamoto D, Yang HP, Yang SP, Yorke JA, Yoshida K, Zdobnov E, Zhang P, Zhang Y, Zimin AV, Baldwin J, Abdouelleil A, Abdulkadir J, Abebe A, Abera B, Abreu J, Acer SC, Aftuck L, Alexander A, An P, Anderson E, Anderson S, Arachi H, Azer M, Bachantsang P, Barry A, Bayul T, Berlin A, Bessette D, Bloom T, Blye J, Boguslavskiy L, Bonnet C, Boukhgalter B, Bourzgui I, Brown A, Cahill P, Channer S, Cheshatsang Y, Chuda L, Citroen M, Collymore A, Cooke P, Costello M, D'Aco K, Daza R, De Haan G, DeGray S, DeMaso C, Dhargay N, Dooley K, Dooley E, Doricent M, Dorje P, Dorjee K, Dupes A, Elong R, Falk J, Farina A, Faro S, Ferguson D, Fisher S, Foley CD, Franke A, Friedrich D, Gadbois L, Gearin G, Gearin CR, Giannoukos G, Goode T, Graham J, Grandbois E, Grewal S, Gyaltsen K, Hafez N, Hagos B, Hall J, Henson C, Hollinger A, Honan T, Huard MD, Hughes L, Hurhula B, Husby ME, Kamat A, Kanga B, Kashin S, Khazanovich D, Kisner P, Lance K, Lara M, Lee W, Lennon N, Letendre F, LeVine R, Lipovsky A, Liu X, Liu J, Liu S, Lokyitsang T, Lokyitsang Y, Lubonja R, Lui A, MacDonald P, Magnisalis V, Maru K, Matthews C, McCusker W, McDonough S, Mehta T, Meldrim J, Meneus L, Mihai O, Mihalev A, Mihova T, Mittelman R, Mlenga V, Montmayeur A, Mulrain L, Navidi A, Naylor J, Negash T, Nguyen T, Nguyen N, Nicol R, Norbu C, Norbu N, Novod N, O'Neill B, Osman S, Markiewicz E, Oyono OL, Patti C, Phunkhang P, Pierre F, Priest M, Raghuraman S, Rege F, Reyes R, Rise C, Rogov P, Ross K, Ryan E, Settipalli S, Shea T, Sherpa N, Shi L, Shih D, Sparrow T, Spaulding J, Stalker J, Stange-Thomann N, Stavropoulos S, Stone C, Strader C, Tesfaye S, Thomson T, Thoulutsang Y, Thoulutsang D, Topham K, Topping I, Tsamla T, Vassiliev H, Vo A, Wangchuk T, Wangdi T, Weiand M, Wilkinson J, Wilson A, Yadav S, Young G, Yu Q, Zembek L, Zhong D, Zimmer A, Zwirko Z, Jaffe DB, Alvarez P, Brockman W, Butler J, Chin C, Gnerre S, Grabherr M, Kleber M, Mauceli E, MacCallum I, Drosophila 12 Genomes Consortium. (2007). Evolution of genes and genomes on the Drosophila phylogeny.. Nature.

[R8] Gramates L Sian, Agapite Julie, Attrill Helen, Calvi Brian R, Crosby Madeline A, dos Santos Gilberto, Goodman Joshua L, Goutte-Gattat Damien, Jenkins Victoria K, Kaufman Thomas, Larkin Aoife, Matthews Beverley B, Millburn Gillian, Strelets Victor B, Perrimon Norbert, Gelbart Susan Russo, Agapite Julie, Broll Kris, Crosby Lynn, dos Santos Gil, Falls Kathleen, Gramates L Sian, Jenkins Victoria, Longden Ian, Matthews Beverley, Seme Jolene, Tabone Christopher J, Zhou Pinglei, Zytkovicz Mark, Brown Nick, Antonazzo Giulia, Attrill Helen, Garapati Phani, Goutte-Gattat Damien, Larkin Aoife, Marygold Steven, McLachlan Alex, Millburn Gillian, Öztürk-Çolak Arzu, Pilgrim Clare, Trovisco Vitor, Calvi Brian, Kaufman Thomas, Goodman Josh, Krishna Pravija, Strelets Victor, Thurmond Jim, Cripps Richard, Lovato TyAnna, the FlyBase Consortium (2022). FlyBase: a guided tour of highlighted features. Genetics.

[R9] Graveley BR, Brooks AN, Carlson JW, Duff MO, Landolin JM, Yang L, Artieri CG, van Baren MJ, Boley N, Booth BW, Brown JB, Cherbas L, Davis CA, Dobin A, Li R, Lin W, Malone JH, Mattiuzzo NR, Miller D, Sturgill D, Tuch BB, Zaleski C, Zhang D, Blanchette M, Dudoit S, Eads B, Green RE, Hammonds A, Jiang L, Kapranov P, Langton L, Perrimon N, Sandler JE, Wan KH, Willingham A, Zhang Y, Zou Y, Andrews J, Bickel PJ, Brenner SE, Brent MR, Cherbas P, Gingeras TR, Hoskins RA, Kaufman TC, Oliver B, Celniker SE (2010). The developmental transcriptome of Drosophila melanogaster.. Nature.

[R10] Grewal SS (2008). Insulin/TOR signaling in growth and homeostasis: a view from the fly world.. Int J Biochem Cell Biol.

[R11] Gruys ML, Sharp MA, Lill Z, Xiong C, Hark AT, Youngblom JJ, Rele CP, Reed LK (2025). Gene model for the ortholog of Glys in Drosophila simulans.. MicroPubl Biol.

[R12] Hietakangas V, Cohen SM (2009). Regulation of tissue growth through nutrient sensing.. Annu Rev Genet.

[R13] Jenkins VK, Larkin A, Thurmond J, FlyBase Consortium (2022). Using FlyBase: A Database of Drosophila Genes and Genetics.. Methods Mol Biol.

[R14] Kent WJ, Sugnet CW, Furey TS, Roskin KM, Pringle TH, Zahler AM, Haussler D (2002). The human genome browser at UCSC.. Genome Res.

[R15] Kikkawa Hideo (1938). Studies on the genetics and cytology ofDrosophila ananassae. Genetica.

[R16] Larkin A, Marygold SJ, Antonazzo G, Attrill H, Dos Santos G, Garapati PV, Goodman JL, Gramates LS, Millburn G, Strelets VB, Tabone CJ, Thurmond J, FlyBase Consortium. (2021). FlyBase: updates to the Drosophila melanogaster knowledge base.. Nucleic Acids Res.

[R17] Lawson ME, McAbee M, Lucas RA, Tanner S, Wittke-Thompson J, Pelletier TA, Ozsoy Z, Sterne-Marr R, Rele CP, Reed LK (2024). Gene model for the ortholog of Ilp5 in Drosophila ananassae.. MicroPubl Biol.

[R18] Markow TA and O’Grady P. (2005) Drosophila: A guide to species identification and use. 978-0-12-473052-6

[R19] McLaughlin S, Dixon JE (1993). Alternative splicing gives rise to a nuclear protein tyrosine phosphatase in Drosophila.. J Biol Chem.

[R20] Mudge JM, Harrow J (2016). The state of play in higher eukaryote gene annotation.. Nat Rev Genet.

[R21] Myers A, Hoffman A, Natysin M, Arsham AM, Stamm J, Thompson JS, Rele CP, Reed LK (2024). Gene model for the ortholog Myc in Drosophila ananassae.. MicroPubl Biol.

[R22] Navarro Gonzalez J, Zweig AS, Speir ML, Schmelter D, Rosenbloom KR, Raney BJ, Powell CC, Nassar LR, Maulding ND, Lee CM, Lee BT, Hinrichs AS, Fyfe AC, Fernandes JD, Diekhans M, Clawson H, Casper J, Benet-Pagès A, Barber GP, Haussler D, Kuhn RM, Haeussler M, Kent WJ (2021). The UCSC Genome Browser database: 2021 update.. Nucleic Acids Res.

[R23] Raney BJ, Dreszer TR, Barber GP, Clawson H, Fujita PA, Wang T, Nguyen N, Paten B, Zweig AS, Karolchik D, Kent WJ (2013). Track data hubs enable visualization of user-defined genome-wide annotations on the UCSC Genome Browser.. Bioinformatics.

[R24] Rele Chinmay P., Sandlin Katie M., Leung Wilson, Reed Laura K. (2023). Manual annotation of Drosophila genes: a Genomics Education Partnership protocol. F1000Research.

[R25] Singh BN, Yadav JP (2015). Status of research on Drosophila ananassae at global level.. J Genet.

[R26] Singh BN (2010). Drosophila ananassae: a good model species for genetical, behavioural and evolutionary studies.. Indian J Exp Biol.

[R27] Sturtevant AH (1939). On the Subdivision of the Genus Drosophila.. Proc Natl Acad Sci U S A.

[R28] Tchankouo-Nguetcheu S, Udinotti M, Durand M, Meng TC, Taouis M, Rabinow L (2014). Negative regulation of MAP kinase signaling in Drosophila by Ptp61F/PTP1B.. Mol Genet Genomics.

[R29] Tello-Ruiz MK, Marco CF, Hsu FM, Khangura RS, Qiao P, Sapkota S, Stitzer MC, Wasikowski R, Wu H, Zhan J, Chougule K, Barone LC, Ghiban C, Muna D, Olson AC, Wang L, Ware D, Micklos DA (2019). Double triage to identify poorly annotated genes in maize: The missing link in community curation.. PLoS One.

[R30] Willoughby LF, Manent J, Allan K, Lee H, Portela M, Wiede F, Warr C, Meng TC, Tiganis T, Richardson HE (2017). Differential regulation of protein tyrosine kinase signalling by Dock and the PTP61F variants.. FEBS J.

[R31] Wu CL, Buszard B, Teng CH, Chen WL, Warr CG, Tiganis T, Meng TC (2011). Dock/Nck facilitates PTP61F/PTP1B regulation of insulin signalling.. Biochem J.

